# Osteoarthritis: a common disease that should be avoided in the athletic horse’s life

**DOI:** 10.1093/af/vfac026

**Published:** 2022-06-14

**Authors:** Raquel Yvonne Arantes Baccarin, Sarah Raphaela Torquato Seidel, Yara Maria Michelacci, Paula Keiko Anadão Tokawa, Tiago Marcelo Oliveira

**Affiliations:** Department of Internal Medicine, Faculdade de Medicina Veterinária e Zootecnia, Universidade de São Paulo, São Paulo, Brazil; Department of Internal Medicine, Faculdade de Medicina Veterinária e Zootecnia, Universidade de São Paulo, São Paulo, Brazil; Department of Biochemistry, Escola Paulista de Medicina, Universidade Federal de São Paulo, São Paulo, Brazil; Department of Internal Medicine, Faculdade de Medicina Veterinária e Zootecnia, Universidade de São Paulo, São Paulo, Brazil; Department of Internal Medicine, Faculdade de Medicina Veterinária e Zootecnia, Universidade de São Paulo, São Paulo, Brazil

**Keywords:** athletic horses, osteoarthritis, performance, welfare

**Implications**

Osteoarthritis progress involves considerable damage to the affected joint, directly impacting health, welfare, and performance of athletic horses;

Knowledge of the disease’s pathways are essential to determine the treatment approach;

Accurate and early diagnosis is important to establish the severity and improve the prognosis;

Physical therapy and rehabilitation programs are crucial to improve the return of the athletic abilities of the horse.

## Introduction

High-performance sports require athletes to walk a narrow line that separates maximum performance from injury. Athletes often exceed the physiological limit to achieve the best results, bringing a great challenge to physical trainers, physiotherapists, and physicians. In equine science, this scenario is not different; the veterinarian must have a deep knowledge of the anatomy and physiology of different structures to prevent injuries that can be caused during high-level exercise practice.

One of the main causes of lameness and, therefore, failure in equine athletes is joint disease, and osteoarthritis (OA) usually follows the athletic career of many horses in different sports (Murray, 2006). Just as soccer players suffer from knee injuries and tennis players live with joint changes in the wrist, each sport modality in horses usually presents the involvement of some joints most used when they play that sport. Osteoarthritis of the metacarpophalangeal (fetlock) joint is very common in jumping horses due to the overload received when the horse lands, while the tarsal (hock) joints are usually the seat of OA in horses that dispute the western competitive events, due to abrupt stops and changes in direction required by the exercises (De Sousa et al., 2017).

Exercise is necessary to maintain joint homeostasis and plays a crucial role in the physiological maturation of joints in young horses. Moller and van Weeren (2017) described three main mechanisms by which exercise can influence joint homeostasis: (1) the direct effect of mechanical impact on cartilage tissue integrity, (2) the indirect influence on joint tissue metabolism, and (3) the influence of exercise on joint circulation. Research on the effects of exercise on cartilage has shown that, as occurs in bone, cartilage tends to weaken without regular loading, but unlike bone, cartilage does not appear to thicken even in elite athletes, although some studies do not support this observation (Gahunia and Pritzker, 2012).

Moller and van Weeren (2017) also observed that physical overload (for example, too much exercise or concussion) disrupts the integrity of joint structures, resulting in inflammatory responses. Such disturbances of joint homeostasis negatively affect chondrocyte vitality and response and can eventually lead to irreparable damage to the articular cartilage. However, the frequency and intensity of exercise could stimulate the recovery of joint homeostasis and joint function by stimulating proteoglycan synthesis and promoting circulation within the various joint components (Moller and van Weeren, 2017). In summary, both pre-OA and adaptive changes can occur without obvious clinical signs in horse athletes, and several ways to monitor the evolution and maturation of joints to avoid the occurrence of injuries can be employed (Baccarin et al., 2014; Yamada et al., 2020).

## Changes in the Remodeling of Cartilage and Osteoarthritis

All types of joint injury can lead to the development of OA, which is clinically characterized by heat, pain, swelling, and decreased range of motion in the affected joints. OA is a complex, multifaceted disorder that manifests itself in cartilage degradation (loss of its components as type II collagen and proteoglycans), increase in cartilage cell metabolism (chondrocyte synthetic activity, proliferation, and apoptosis), synovial inflammation, hyperplasia and hypertrophy, and bone changes (subchondral sclerosis and osteophyte formation) (Ribitsch et al., 2021). Although OA is a disease of the entire joint, the destruction of cartilage is a central and irreversible step in the process.

### Cartilage

**Figure 1.**
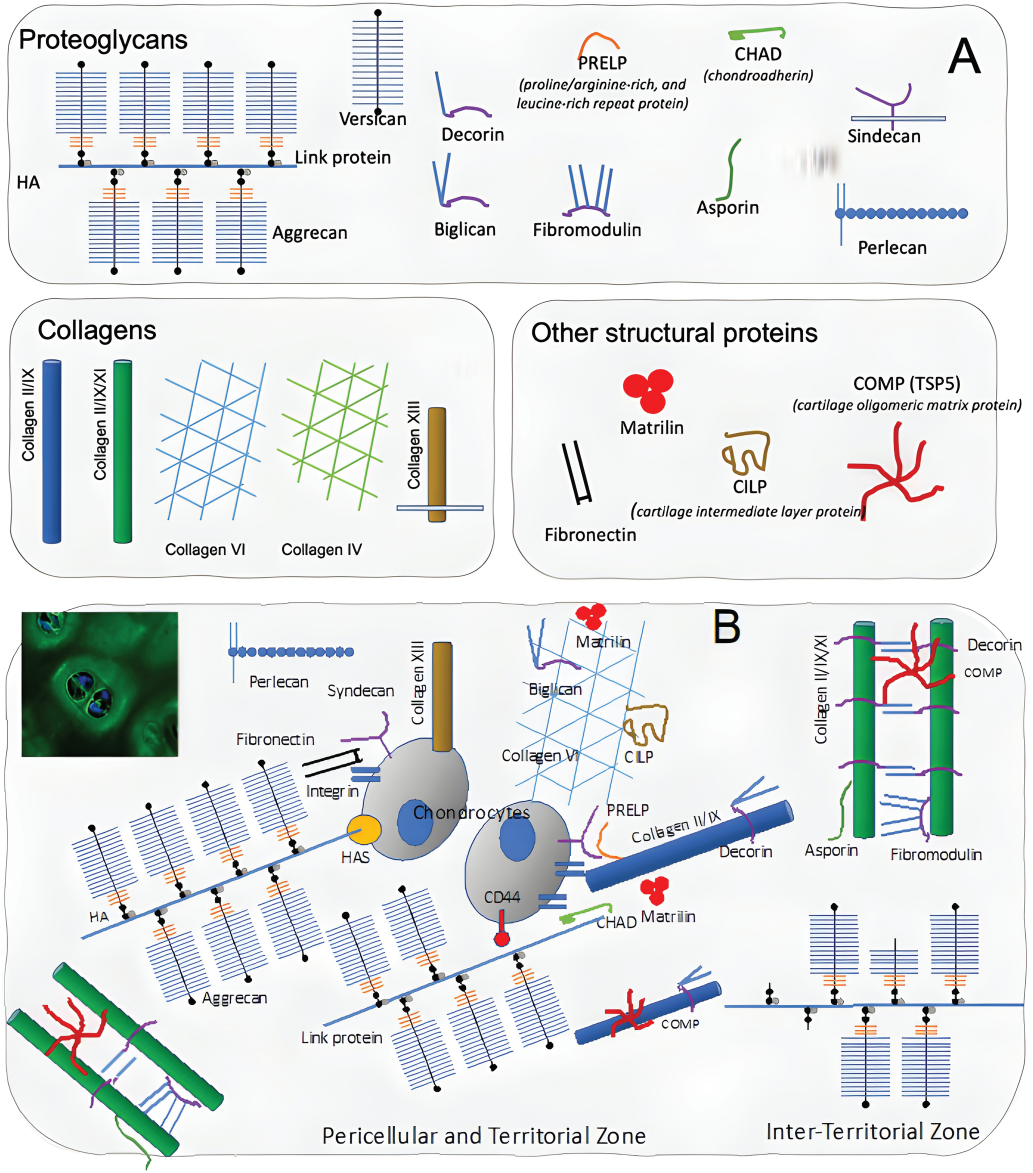
**Articular cartilage ECM: components (A) and organization (B).** There are differences in the ECM composition at the pericellular and “territorial zone”, closer to the cells, in comparison to the “interterritorial zone”. The interterritorial zone is composed of a fibrillar collagen network, consisting primarily of type II collagen fibrils with types XI and IX collagens integrated. In contrast, the territorial matrix contains type VI collagen microfibrils, but little fibrillar collagen.

Cartilage is an avascular, extracellular matrix (ECM)-rich tissue that consists of one cell type, the chondrocytes, and the ECM they produce. The main components of cartilage ECM are collagens, proteoglycans, and other noncollagen structural proteins. They interact with each other, creating a multicomponent structural meshwork that surrounds and hosts the chondrocytes. Extracellular matrix molecules also interact with chondrocytes through different cell surface receptors (Figure 1).

#### Cartilage collagens.

Collagens are the most abundant ECM proteins in adult articular cartilage. Type II collagen, the primary cartilage collagen, forms fibrils that provide tensile strength, but many other collagen types are also present in cartilage (Eyre, 2004). Type IV and type VI collagens are found around chondrocytes, in the pericellular matrix, and can be linked to the type II collagen fibrils via matrilin-4 and biglycan. Types IX and XII collagens do not form fibrils by themselves but are associated with the surface of various fibrils. Type XI collagen is retained at the chondrocyte surface and involved in the organization of the pericellular matrix via interaction with cartilage proteoglycans (Eyre, 2004) (Figure 1).

#### Cartilage proteoglycans.

Proteoglycans are the major noncollagenous proteins found in the articular cartilage ECM. Two main proteoglycan families are present in the cartilage ECM: hyalectans (aggrecan and versican) and small leucine-rich proteins/proteoglycans (SLRPs). Other proteoglycans that belong to other families, such as perlecan and type IX collagen, are also present.

Aggrecan is the main cartilage proteoglycan. It forms a hydrated gel structure that confers the cartilage its load-bearing properties. Aggrecan molecules form large aggregates in the ECM and are concentrated in the cartilage territorial zone (Figure 1). Each aggregate is composed of a central filament of hyaluronic acid with up to 100 aggrecan molecules radiating from it, and each interaction is stabilized by a link protein (Morgelin et al., 1994).

Small leucine-rich proteins/proteoglycans, including decorin, biglycan, fibromodulin, proline/arginine-rich, leucine-rich repeat protein, and chondroadherin (CHAD) are also important for proper cartilage function (Roughley et al., 1996). All of them can bind to collagen, and chondroadherin and PRELP are also able to link members of the syndecan family of cell surface proteoglycans. Asporin is another small leucine-rich proteins/proteoglycans expressed in cartilage and may play different roles in the pathogenesis of OA (Xu et al., 2015) (Figure 1).

#### Noncollagen proteins.

Other noncollagen structural proteins, including cartilage oligomeric matrix protein (COMP), matrilin, chondromodulin, and cartilage intermediate layer protein (CILP), are also present in the cartilage ECM; and COMP is a major noncollagenous protein in joint cartilage (Figure 1).

### Pathobiological processes of OA

In performance horses, two basic pathobiological processes of OA can be considered: inflammation of the synovial membrane and fibrous joint capsule (synovitis and capsulitis), and physical and/or biochemical damage to the articular cartilage and bone (McIlwraith et al., 2016).

The synovium is involved early in the OA process. However, it remains unclear whether the changes that occur in the OA synovial membrane occur first, or whether they are the result of cartilage degradation or lesions of the subchondral bone. Several cell types may be responsible for synovial inflammation, but studies have shown that much of the inflammation in the OA joint are attributed to synovial proinflammatory macrophages (Menarim et al., 2020). Catabolic and proinflammatory mediators, such as cytokines (interleukin-1β, interleukin-6, and tumor necrosis factor α), nitric oxide, prostaglandin E_2_, and neuropeptides, produced by the inflamed synovium further stimulate cartilage ECM degradation. Cartilage alteration amplifies synovial inflammation, creating a vicious circle (Sellam and Berenbaum, 2010).

**Figure 2.**
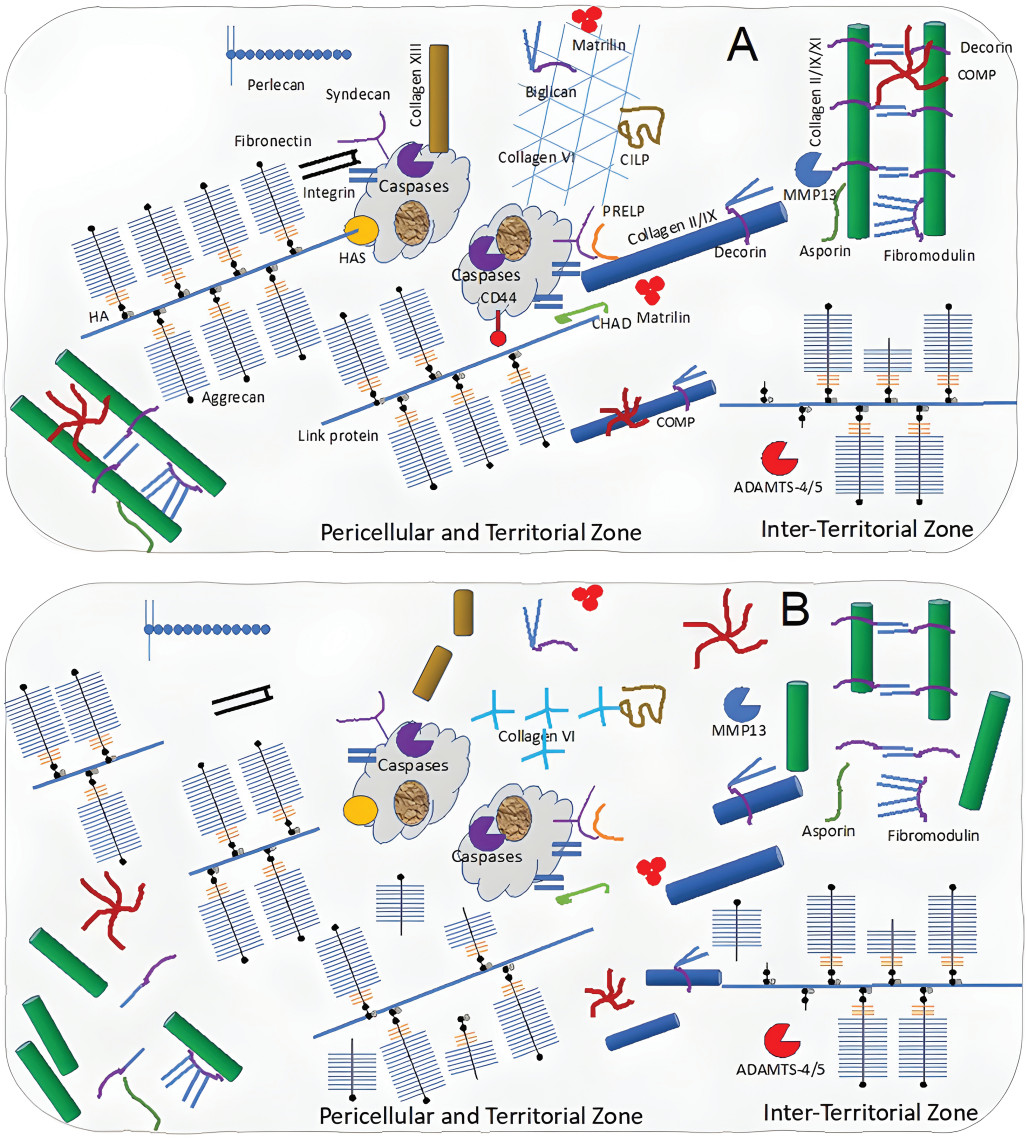
**Changes in cartilage organization in OA.** The depletion of aggrecan, as the result of proteolytic cleavage of the aggrecan core protein by proteases, is a key event in OA. IL-1β creates an inflammation microenvironment that leads to the loss of ECM components, chondrocyte hypertrophy, and terminal differentiation. Chondrocytes begin to express Runt-related transcription factor (RUNX2), vascular endothelial growth factor (VEGF), collagen X, and MMP13. Calcification of the ECM around chondrocytes follows this shift towards hypertrophy, leading to subchondral bone sclerosis.

Cartilage ECM degradation can also be result of direct physical damage to the articular cartilage and subchondral bone. Under normal conditions, articular chondrocytes and subchondral osteoblasts receive mechanical loads, and are able to withstand them without being damaged or producing inflammatory molecules. Eventually, homeostasis cannot sufficiently compensate for the mechanical load and strain on the body, and the cartilage loss begins. Throughout the initial period of OA, there is increased proliferation and enhanced tissue remodeling, with the synthesis of new cartilage ECM components to maintain structural integrity and homeostasis. However, an imbalance between synthesis and breakdown may lead to the secretion of proinflammatory cytokines, matrix metalloproteinases, and aggrecanases (ADAMTS-1, 4, and 5) which cause cartilage degradation (Goldring and Goldring, 2010) (Figure 2). In addition, exposure to inflammatory and oxidative mediators enhances premature stress-induced senescence and aging of chondrocytes (Ribitsch et al., 2021).

## Diagnosis of Osteoarthritis in Horses

In the clinical setting, the diagnosis of OA is usually achieved through the association of clinical and imaging findings. Radiographic examination is generally the most employed imaging tool to assess bone changes in joints, such as new irregular bone formation, development defects, reduction of joint space, and others (Baxter, 2020). However, its diagnostic value is limited as it cannot detect soft tissue or subtle bone changes (Barrett et al., 2018).

Ultrasonographic examination is usually used in association with radiographs, as it can detect early osteochondral irregularities, as well as changes in joint soft tissues, including articular cartilage (Baxter, 2020). The power Doppler tool can provide further information on increases in vascularization of the joint synovium and capsule, reflecting the inflammation of such tissues (Yamada et al., 2020). Strain elastosonography has recently been reported as an additional tool for ultrasonographic imaging, aiming to detect reduced elasticity (increase in stiffness) in the joint capsule (Straticò et al., 2021).

Arthroscopy allows direct visualization and subjective evaluation of synovial structures. In osteoarthritic joints, the assessment of synovial membrane shape during surgical arthroscopy showed a high correlation with their histological aspects, allowing it to be a good indicator of the ongoing inflammatory process (Agreste et al., 2021). Further information, such as surface integrity, thickness and stiffness of articular cartilage, and integrity of ligaments, can also be assessed (McIlwraith et al., 2014). However, arthroscopy is not sufficient to identify early degenerative changes in cartilage structure and composition. Recent studies have investigated arthroscopic near-infrared spectroscopy technique as a diagnostic tool to evaluate cartilage composition and biomechanical properties, as well as subchondral bone architecture (Sarin et al., 2019).

Magnetic resonance imaging (MRI) has been used increasingly in equine sports medicine and allows a detailed examination of different synovial structures. High-field systems are usually required for a better assessment of lesions related to articular cartilage, as they allow early identification of changes in cartilage morphology, such as its thickness, volume, and surface area in the joint. Other MRI findings, such as subchondral bone lysis/sclerosis and increased fluid signals in the trabecular and subchondral bone and bone marrow changes, are usually associated with articular cartilage injury and OA progression (Nelson et al., 2018). Contrast can be applied to improve the outlining of cartilage and other soft tissues, increasing diagnostic sensitivity (Suarez Sanches-Andrade et al., 2018). In addition to morphological evaluation, the development of quantitative MRI techniques has allowed the identification of biochemical and biomechanical changes in cartilage, allowing the diagnosis of early degenerative conditions that precede morphological changes (Nelson et al., 2018). Extracellular matrix components such as glycosaminoglycans, collagen, and water are usually highlighted in these techniques, which have shown promising results in detecting early changes in OA (Kajabi et al., 2021).

Conventional computed tomography (CT) allows more accurate evaluation of the same bone changes found on radiographs, but has poorer soft tissue imaging capacity (Baxter, 2020). To eliminate such limitations, contrast-enhanced CT techniques have been developed involving the application of iodinated contrast media (ICM), allowing the acquisition of critical information from joint soft tissues (McIlwraith et al., 2018). Recent techniques with ICM aiming to detect early cartilage injury allow for further quantitative evaluation of articular cartilage, which includes biochemical (glycosaminoglycan content) and biomechanical (mechanical stiffness) changes, similar to quantitative MRI assessment (Nelson et al., 2018).

In addition to imaging techniques, the measurement of OA biomarkers has also been used, usually in research. Studies have focused on measuring the concentrations or expression of markers involved in the pathological pathways of OA and metabolism of tissues (McIlwraith et al., 2018). Proinflammatory cytokines, such as IL-1 and IL-6, enzymes, such as MMP-2, -3, -9, -13, ADAMTS-5, concentrations of lubricin, products from collagen, and other cartilage molecule degeneration, such as CTX-II, COMP, COMP neoepitope, chondroitin sulfate, and glycosaminoglycans, are some examples of measured biomarkers in the synovial fluid to detect OA processes in horses (Ma et al., 2017; Watkins and Reesink, 2020). Some biomarkers measured in equine serum have also been described as useful in detecting early OA (CS846, CPII, glycosaminoglycans, C1, and 2C) (Frisbie et al., 2008). Chondroitin sulfate measured in the urine of horses has also been used as a useful marker of cartilage degradation (Baccarin et al., 2012).

## Approach to Joint Treatment

There is no single therapy that is capable of completely stopping OA progression and restoring hemostasis to the articular environment; therefore, it is a common practice to use two or more treatments in attempting to improve the prognosis and the possibility of returning to sports with similar performance.

### Anti-inflammatory drugs

The use of anti-inflammatory drugs remains a regular practice among equine veterinarians in OA cases. Nonsteroidal anti-inflammatory drugs (NSAIDs) are often used systemically to improve clinical symptoms, such as pain and lameness. Their sustained use must be carefully evaluated to avoid adverse effects. Phenylbutazone is one of the most commonly used NSAIDs (Goodrich and Nixon, 2006); however, COX-2–selective NSAIDs, such as meloxicam and firocoxib, have a superior gastrointestinal safety profile. Regardless of the anti-inflammatory drug chosen, its use is not an ideal long-term solution for the management of OA.

Corticosteroids are usually administered intra-articular, which presents a powerful anti-inflammatory action. Betamethasone acetate, methylprednisolone acetate, and triamcinolone acetonide are some of the most widely used intra-articular corticosteroids. Different products and corticosteroid doses vary in their benefits versus deleterious effects (McIlwraith et al., 2016; Pontes-Quero et al., 2019). Similar to NSAIDs, sustainable corticosteroid treatment also requires prudent use, in this case, due to its deleterious effects on articular cartilage (Neuenschwander et al., 2019). Studies have confirmed that relief from symptoms using intra-articular corticosteroids is short-lived, and no other benefits are seen half a year later. To relieve symptoms, intra-articular corticosteroids should not be injected more than four times a year (Zhang et al., 2020). In our clinical experience, cartilage loss and subchondral bone exposure are common findings on arthroscopy in joints that received up to three injections of intra-articular corticosteroids in a period of 1 year in horses kept in sports activity.

Although dimethylsulfoxide (DMSO) is not classified as anti-inflammatory, it has analgesic and anti-inflammatory properties and is commonly applied in the equine routine in joint lavages as an additional treatment for synovium inflammation. In an experimental study, joint lavage using a diluted DMSO solution decreased lameness, inflammatory cell count, PGE_2_, and proinflammatory cytokines (IL-1β and IL-6) compared to solution without DMSO (Sotelo et al., 2020).

### Viscosupplementation

Since the early 1970s, intra-articular hyaluronic acid has been used to treat synovitis and OA in racehorses. However, the efficacy of hyaluronic acid in the treatment of OA remains controversial. The rationale for intra-articular hyaluronic acid injection is, mainly, that it improves viscosity of the synovial fluid and increases secretion of endogenous hyaluronic acid improving the homeostasis of the joint (Zhang et al., 2020).

Some studies have pointed out that intra-articular injection of hyaluronic acid alone cannot substantially reduce lameness (Contino, 2018), however the degree of anti-inflammatory, immunomodulatory, analgesic, and anti-OA effects are determined by the molecular weight of hyaluronic acid, route of administration, and number of intra-articular injections (Zhang et al., 2020). Nowadays, some commercial viscosupplements combine hyaluronic acid and other bioactive principles, the most common being different antioxidant molecules such as mannitol and sorbitol (Pontes-Quero et al., 2019).

Hyaluronic acid is mainly indicated for mild to moderate levels of synovitis associated with equine OA, and it has limitations in treating severe synovitis or OA. It presents an alternative therapeutic approach to intra-articular corticosteroids, especially for acute synovitis treatment (Neuenschwander et al., 2019). Despite this, literature has demonstrated a further improvement of short term pain relief when adding intra-articular corticosteroids to viscosupplements (Pontes-Quero et al., 2019).

Intra-articular hyaluronic acid injection is the most efficacious manner to achieve the maximum drug concentration in the joint, but the risks of joint infection and the desire to treat multiple joints at the same time led to the development of oral and intravenous preparations. There is only one study documenting efficacy of intravenous hyaluronic acid and no objective data on the absorption or efficacy of oral hyaluronic acid products was presented (Goodrich and Nixon, 2006).

### Polysulfated polysaccharides

Polysulfated glycosaminoglycans, pentosan polysulfate, and CS belong to a group of polysulfated polysaccharides used in OA treatment. Polysulfated glycosaminoglycans act to sustain and/or promote chondrocyte metabolic activity, inhibiting the detrimental effects of proinflammatory cytokines and PGE_2_ on articular cartilage, slowing OA progression, and stimulating glycosaminoglycan and hyaluronic acid production (McIlwraith et al., 2016). Polysulfated glycosaminoglycans are primarily CS, and there is evidence that CS elicits an anti-inflammatory effect on synovium and chondrocyte levels (da Cunha et al., 2017). Combined use of CS and glucosamine is believed to boost chondroprotective effects. A hyaluronic acid, sodium chondroitin sulfate, and N-acetyl-d-glucosamine combination product has been used by equine practitioners for the treatment of OA although there are no peer-reviewed scientific articles on the subject. Several *in vivo* studies reveal that pentosan polysulfate inhibits various processes that induce degeneration of the articular cartilage matrix (McIlwraith et al., 2016).

Nowadays, there are many oral compounds sold as supplements that contain varying concentrations of glucosamine, CS, methylsulfonylmethane, unsaponifiable avocado and soybean extracts, polyunsaturated omega-3 fatty acids, type II collagen, among others which can be used to slow OA progression (Goodrich and Nixon, 2006).

### Regenerative therapy

The majority of regenerative therapy substances are considered orthobiologics, which means that they originate from components naturally found in tissues from the animal itself or a compatible donor. These biological strategies can be divided into two groups based on their source, cell or blood-derived, and are based on the biological and natural capacity of the organism to heal itself (Monteiro et al., 2015). Orthobiologics are becoming increasingly prevalent in the treatment of equine OA.

#### Autologous-conditioned serum and autologous-protein solution.

Autologous-conditioned serum is produced using commercial kits (IRAP I: Orthokine; IRAP II: Arthrex) using horse’s blood. This methodology allows the product to markedly increase the IL-1 receptor antagonist (IL-1ra) and can also increase other components such as IL-10, growth factor IGF-1, and TGF-β. Furthermore, it has been shown that IRAP reduces oxidative stress in cultured cells and reduces hyaluronic acid breakdown in synovial fluid in osteochondritis joints (Machado et al., 2019). Autologous-protein solution is another commercial product (Pro-Stride, Biomet Biologics) with a different methodology that concentrates anti-inflammatory factors, and significantly increases the number of leukocytes. These products present a potent anti-inflammatory action, noticeably diminishing clinical signs (Bogers, 2018).

#### Platelet-rich plasma.

Platelet-rich plasma is produced from whole blood that results in an enriched platelet product. There are several possible methodologies for its production, causing the effects of platelet-rich plasma to vary greatly. The solution provides growth factors that upregulate glycosaminoglycans and collagen synthesis and inhibit catabolic and inflammatory activity. Clinical amelioration of symptoms and return to athletic performance have been reported in equine OA treated with platelet-rich plasma or platelet lysate (Garbin and Olver, 2020).

#### Mesenchymal stem cells.

Mesenchymal stem cells (MSCs) are defined as multiprogenitor cells that are able to self-replicate and mature into distinct cell types, such as chondrocytes. Bone marrow, adipose tissue, synovium, and synovial fluid are among the possible sources of MSCs for intra-articular use. However, there is a lack of consensus on the best cellular source, number of cells, number of infiltrations, interval time between them, use of autologous (from the same animal) or allogenic (from a different animal) cells, and time for initiation of treatment; despite that, MSCs have immunomodulatory and anti-inflammatory effects. The outcomes reported showed an improvement in clinical signs, cartilage appearance, articular environment, and return to previous performance (Zayed et al., 2018).

### Other therapies

Polyacrylamide hydrogel is used with promising results; however, this treatment warrants further investigation. Extracorporeal shock wave therapy is particularly useful for the treatment of OA in low-motion joints (pasterns and hocks) and enthesopathies, which can occur at the joint capsule insertion (Contino, 2018). Bisphosphonates (tiludronate and clodronate) alter bone metabolism, and their use is recommended in cases where there is too much bone resorption. Tiludronate appears to inhibit the radiographic progression of OA in the high-motion joints of racehorses (Bertuglia et al., 2021). There is only one study documenting safety of intra-articular use of the medical ozone in horses in the current literature (Vendruscolo et al., 2018). Further studies should be conducted to understand the benefits of intra-articular ozone therapy.

### Surgical intervention

Arthroscopy to remove debris and flush the joint with medicated fluids is considered palliative surgical care, whereas the use of marrow stimulation techniques, osteochondral grafting, autologous chondrocyte implantation, and augmentation with MSCs are some of the reparative or restorative techniques (Johnson and Frisbie, 2016). It is important to note that any of these surgical procedures can be associated with other intra-articular therapies to achieve better results.

When all treatments fail to return the horse to athletic career or comfortable use of the limb, arthrodesis (surgical fusion of a joint, resulting in bony ankylosis) is a final option for some joints. In horses with osteoarthritis of low-motion joints, such as the proximal interphalangeal or the distal tarsal joints, arthrodesis is performed to return horses to athletic life. In high-motion joints, arthrodesis is performed to improve the horse’s use of the limb, giving the horse long-term comfort rather than returning the horse to performance (Zubrod and Schneider, 2005).

### Rehabilitation

It is expected that the osteoarthritic joint presents a reduction in proprioception, muscular tone, balance, and limited amplitude movement in response to tissue alterations. Therefore, the implementation of complementary medicine is not only aimed at accelerating the return to performance but also at keeping horses in competition.

Many rehabilitative approaches are available to manage specific clinical alterations of OA, but no single one is highly efficacious in all phases of joint healing. Initially, rehabilitation approaches incorporate methods of pain reduction, limiting adverse effects of joint inflammation, restoring joint flexibility and stability, and maximizing strength and coordination of the affected limb. Long-term approaches include the return to previous levels of exercise and performance, and prevention or recurrence of joint injury. The most common rehabilitation approaches used in our clinical practice are listed below.

Thermal therapy is an inexpensive and feasible method. Cold application decreases pain and swelling, whereas heat application can reduce joint stiffness. Cold therapy should be applied for 15 to 20 minutes every 2 to 3 hours during the first 48 hours after acute injury. It may be indicated for up 10 to 14 days postinjury, depending on the severity and type of injury. Heat therapy is generally contraindicated for acute inflammation. Hydrotherapy relies on some water properties and their therapeutic effects on locomotor system, comprising pressure, viscosity, drag force, and buoyancy properties to relieve pain, reduction of axial forces supported by joints and soft tissues, improvement of joint range of motion, control of edema, and stimulation of blood circulation (Muñoz et al., 2019; Atalaia et al., 2021).

Laser therapy and therapeutic ultrasound are noninvasive approaches that can be applied using different intensities and frequencies depending on the timing of the therapy and the depth of the targeted tissue (Atalaia et al., 2021). Laser therapy is mainly used to reduce joint pain, effusion, and periarticular swelling. Its anti-inflammatory effect occurs via stimulation of prostaglandins, leading to vasodilation. Regarding equine athlete, its use can be combined with PRP at beginning, and maintained during a rehabilitation protocol, sustaining its analgesic effects and preventing recidivism. Therapeutic ultrasound has a good penetration, and increases temperature, cellular metabolism, and oxygen demand, allowing cellular infiltration to the region including inflammatory components and mast cells. It is well absorbed in high-protein tissues, but is reflected by cartilage and bone, although both present high in protein. The continuous mode provides thermal effects on target tissues, improving joint range of motion when applied on joint capsule. (Schlachter and Lewis, 2016).

**Figure 3.**
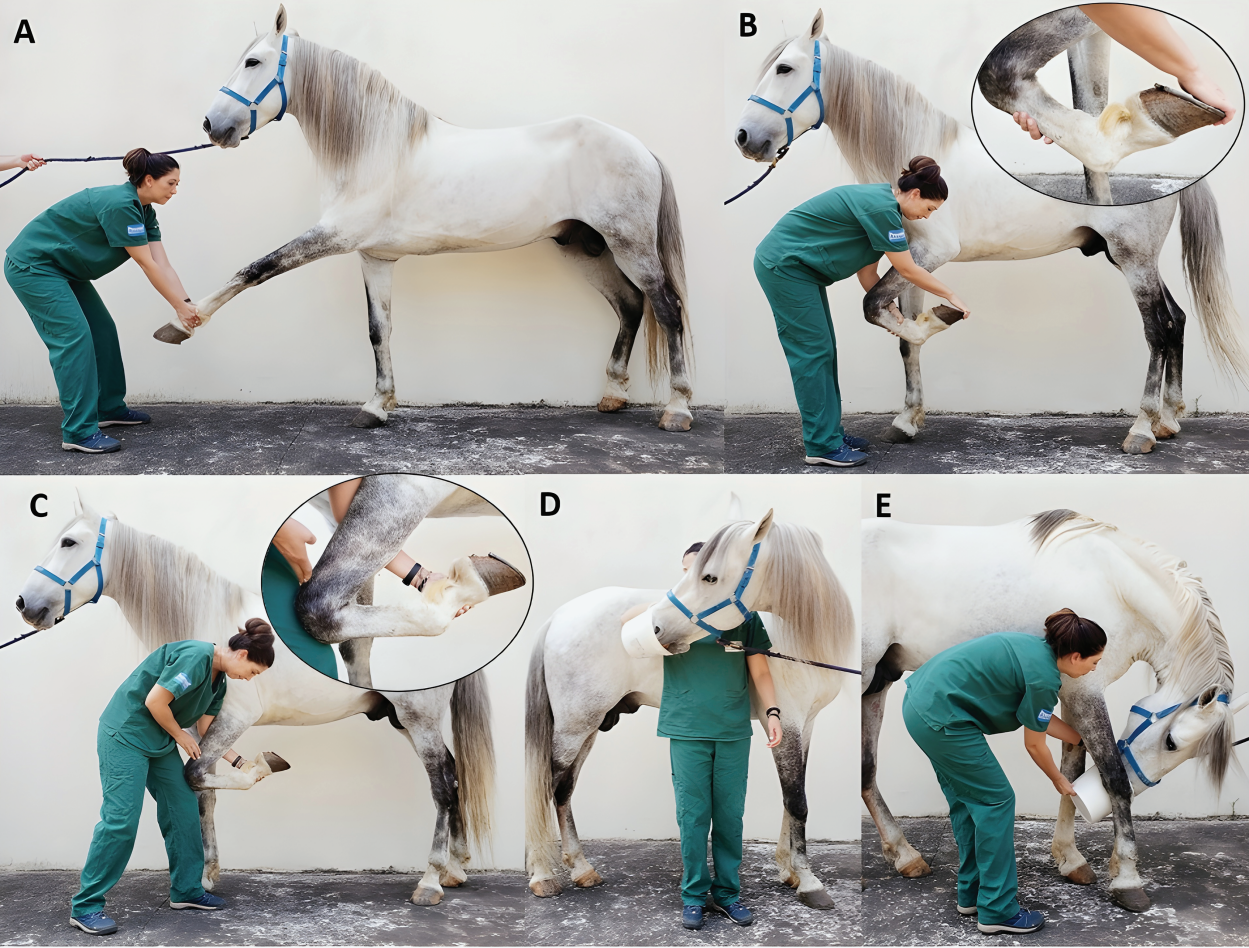
**Manual physio therapy on thoracic limbs**. Passive stretching (A, B, C) with possibility of joint mobilization (B, C); and active stretching of neck (D, E).

Manual therapy involves the use of the hands on the body. Joint mobilization and manipulation are techniques that promote articular movements and are critical in the management of muscular, articular, and neurologic components of selected musculoskeletal injuries in performance horses. They are biomechanically different from each other by the presence of a high-speed impulse, and both can be focused on a specific joint or anatomic region, despite mobilization being often considered a general technique. While joint mobilization is focused on reducing pain, recovering tissue compliance, improving overall tissue mobility, and joint range of motion; manipulation has less focus on the surrounding soft tissues, and is commonly used to address localized pain and joint stiffness. Their application is more effective in the early clinical stages of disease processes versus end-stage disease, but its use is contraindicated in acute episodes of osteoarthritis or in presence of severe articular instability (Haussler, 2016). The association of different types of manual therapy, including passive and active stretching, joint mobilization, and manipulation can result in better outcomes related to joint range of motion and analgesia (Figure 3).

A controlled exercise program is imperative on a rehabilitation program for the equine athlete, which must be well designed and injury-directed in order to enhance the normal tissue reparative response during rehabilitation. It is necessary to have a basic understanding of the normal tissue response after the injury, to create a rehabilitation program not only to enhance the tissue healing but also to prevent further injury and provide a safe return to sport activities. The basic principle on an ideal after-injury rehabilitation program is reducing the force and strain on injured tissue while the normal reparative process evolves. The frequency, amount, and type of exercise needs to be prescribed and adjusted throughout the entire healing process (Davidson, 2016).

In our clinical practice it is common to evaluate each case to formulate the most suitable exercise-controlled program, which may comprehend an initial stall rest, especially if involves a surgical approach, with a subsequent increase on exercise level. It is given to each owner a respective program and instructions to continue the exercise sections even after the hospital discharge, in order to maintain the physical treatment (Table 1).

**Table 1.** Controlled exercise program recommended two weeks after arthroscopy. Every owner receives the schedule and detailed information regarding its execution.

Period	Controlled Exercise	Duration	Frequency
2 weeks	hand walk	10 min	twice a day
2 weeks	hand walk	15 min	twice a day
4 weeks	hand walk trot	15 min 05 min	twice a day
4 weeks	hand walk trot	15 min 10 min	twice a day
After 3 months	gradually increase exercise level to full training		

The benefits of controlled water treadmill exercise have already been observed in horses with induced osteoarthritis, beginning 15 days after the surgery. The treated group presented improvement in postural control and activity of the stabilizing muscles, increase in joint range of motion, decrease of the compensatory biomechanical alterations resulted from primary joint injury, and improvement of lameness scores when compared to control group (King et al., 2013). It is worth mentioning that exercise on a water treadmill is not recommended in acute inflammatory disorders when located on the distal joints of the limb, due to the significant increase in range of motion (Muñoz et al., 2019).

Other methodologies that can also be applied in the rehabilitation of joint diseases, including electrical stimulations modalities, mechanical agents such kinesiology taping, acupuncture, chiropractic, and osteopathic manipulation, and whole-body vibration plates may be used to improve function and alter the progression of OA in horses. Each case could demand different approaches of physiotherapy and even an association of distinct techniques in order to achieve better outcomes and enable the probability of return to athletic life.

## Osteoarthritis and Animal Welfare

Approximately 60% of equine lameness are related to OA which is often associated with loss of performance and early retirement from athletic careers in many equine sports (Sutton et al., 2009, Oke et al., 2010; van Weeren and Back, 2016). There is no cure for OA. Instead, a multimodal treatment approach is advocated for the management of equine OA so that the horse can continue to perform at the desired level, and therefore, it is a highly burdensome equine health problem (Oke et al., 2010).

Osteoarthritis can affect one or more joints, and as it is an inflammatory process, its main manifestation is joint pain, that is one of the conditions that may interfere with welfare and influence the natural behavior of animals, especially horses. Prolonged (chronic) pain negatively impacts both a horse’s physiology and its mental health, leading to distress (Muir, 2010). Horses suffering from medical conditions with some degree of pain, such as OA and old fractures, may not lie down. However, a period of recumbency is necessary for healthy equine sleep, and its lack can lead to sleep deprivation (Aleman, 2008). Horses that cannot lie down and are REM (Rapid Eyes Movement) sleep-deprived may present progressive weight loss, falls, excessive sleepiness (Bertone, 2006), and possible performance alterations, as reported in human athletes (Martin, 1981). Being a typical prey species, horses rest during approximately 30 NREM (Nonrapid Eyes Movement) sleep periods lasting 3–4 min each throughout the day (Morris, 1988). However, horses must be recumbent to achieve and maintain REM sleep (Williams, 2008).

In an experimental study, it was found that horses with joint pain had more time in recumbency and less time feeding than horses with similar joint pain who received pain-relieving medication (Van Loon, 2010). The severity of OA also appears to alter the recumbency time in horses, as horses with mild OA spent more time in recumbency, while those with severe OA may have been partially sleep-deprived because they lie down less. In an observational study, horses with severe OA had a shorter recumbency time than horses with mild OA during one day of monitoring. The increased recumbency time in mild OA spared the animal’s limb and reduced the overload on the affected limb; however, severe OA decreased the frequency and time of recumbency due to the greater difficulty during joint flexion in the transition from standing to recumbency and vice versa (Oliveira et al., 2022). In face of such difficulties, providing the best environment is essential to ease the horse lying down and sleeping.

The influence of OA on the normal behavior of horses can be measured using several methods. Recumbency time (Greening et al., 2021), facial expressions (Dalla Costa et al., 2014), and heart rate variability (Bachmann et al., 2003) are important tools used to identify changes that harm or influence welfare. Recently, an objective assessment of chronic pain caused by OA, among other diseases, called the Horse Chronic Pain Scale, was proposed (Van Loon, 2021).

Many horses with OA can still be ridden and typically do best when are within consistent activity level. It is important to keep the horse active and healthy, but any changes in horse exercise routine should be gradual, as sudden changes in activity level, either a rapid increase or decrease, can aggravate the OA. When providing pain relief, the horse’s pain level will be lower but their condition remains unchanged. As a result, even if the horse appears better, additional stress on their joints can worsen their overall condition.

Overweight can put extra stress on joints, so obesity is one of the biggest detriments to the management of joint pain. Horses with OA should be kept at a body condition score of 4 or 5 out of 9 (Henneke et al., 1983) to decrease the load on joints. Furthermore, the way a horse bears weight on their hooves can affect joint alignment and wear. Special orthopedic shoeing depending on horse’s condition may be recommend.

The majority of lameness cases in aged horses are also owing to OA. The prevalence of OA is greater than 50% in horses older than 15 years, and in horses over 30 years, it increases to 80–90% (Ireland et al., 2011; Ireland et al., 2012; van Weeren and Back, 2016). However, treatment of OA in aged horses aims mainly to provide-optimal comfort rather than to regain ability to compete (van Weeren and Back, 2016).

Some horses with chronic, severe, and refractory OA, can develop laminitis in the opposite supporting limb, a painful disease that affects the horse’s feet, due to excessive unilateral weight bearing (e.g., if the right forelimb is injured by OA, the horse shifts weight to the left front), worsening the horse’s quality of life. Although OA is not life threatening, it is painful and debilitating, and therefore is an important welfare issue.

## Conclusion

Osteoarthritis is a very common injury that affects performance and welfare of athletic horses. Understanding its causes and developmental pathways is critical for establishing an early diagnosis, adjustments in training, and establishing proper treatment.

Research is continuously advancing to aid horses suffering from such disease, but for now, early injury recognition and diagnosis is essential and treatments integrating different medicines have been an important ally. OA monitoring must be a multidisciplinary effort between veterinarians, riders, trainers, and handlers.

## About the Authors

**Raquel Y. A. Baccarin** is an Associate Professor at the Department of Internal Medicine at the School of Veterinary Medicine and Animal Science at the University of São Paulo—Brazil. She graduated in Veterinary Medicine and had a residency training in Large Animal Surgery at School of Veterinary Medicine and Animal Science at the São Paulo State University—UNESP/Botucatu. She obtained master’s degree in Veterinary Surgery and PhD in Veterinary Science from the University of São Paulo and a Post-Doc training in Molecular Biology at Escola Paulista de Medicina—Federal University of São Paulo (UNIFESP). Along several years she has been on the board of the Brazilian Association of Equine Veterinarian (ABRAVEQ), and she was Regional Ambassador (Latin America) and a board member of the World Equine Veterinary Association (WEVA). She has extensive experience in teaching Equine Medicine and her research interests are focused on joint disorders, regenerative therapies, and rehabilitation of the locomotor system.

**Sarah R. T. Seidel** is a doctorate student at the Department of Internal Medicine of the University of São Paulo and associate professor at Paulista University, Brazil. Her education includes an undergraduate in Veterinary Medicine from Tocantins Federal University, followed by a residence program in Large Animals Internal Medicine and Surgery at University of Brasília, and a master degree in Veterinary Science at the Department of Internal Medicine of the University of São Paulo. Her research interests are focused on regenerative therapies, joint and hoof disorders, and co-culture OA models.

**Yara M. Michelacci** is a Senior Full Professor at Escola Paulista de Medicina, Universidade Federal de São Paulo—UNIFESP, Brazil. She graduated and earned a PhD at Escola Paulista de Medicina and had a Pos-Doc training at the Baylor College of Medicine, TX, USA. She earned the title of “Free Professor” (1996) and received many awards and distinctions, including LAFI Award 1976. She has extensive experience in research and teaching of Biochemistry and Molecular Biology (1976-2021), and her research interests are focused on the glycobiology of the extracellular matrix, aiming especially the proteoglycans. She has studied the changes that occur in extracellular matrix proteoglycans in different physiological and/or experimental conditions, as well as diseases such tumors, osteoarthritis, diabetic nephropathy, and corneal opacification, aiming to better understand the role of these important glycoconjugates.

**Paula K. A. Tokawa** is a master’s student at the Department of Internal Medicine of The University of São Paulo in Brazil. She graduated at The University of Western São Paulo (Brazil) with a sandwich period at The University of Sydney (Australia). She also completed an internship period at an equine hospital (Equicenter Hospital) followed by a residency program in Equine Internal Medicine and Surgery at The University of São Paulo, both in Brazil. Her research focuses on diagnostic tools for osteoarthritic lesions in the equine cervical spine and the clinical relevance of imaging findings.

**Tiago M. Oliveira** is an Assistant Professor at the Department of Internal Medicine, School of Veterinary Medicine and Animal Science of University of São Paulo (FMVZ/USP). He graduated in Veterinary Medicine and obtained master’s degree and PhD in Veterinary Science from the Veterinary Clinical Science (FMVZ/USP). He worked at Jockey Club of São Paulo and he has been teaching Equine Science for 10 years. His research interests are equine exercise physiology, equine sports medicine and equine behavior to improve the horse welfare and performance.
